# Advances in Heparins and Related Research. An Epilogue

**DOI:** 10.3390/molecules23020390

**Published:** 2018-02-12

**Authors:** Jawed Fareed, Peter Bacher, Walter Jeske

**Affiliations:** Loyola University Medical Center, Maywood, IL 60153, USA; h.peter.bacher@gmail.com (P.B.); wjeske@luc.edu (W.J.)

The discovery of heparin in 1916 by Jay McLean, a medical student at Johns Hopkins University, not only provided a universal anticoagulant, but also laid the foundation for the discipline of hemostasis and thrombosis. Much of what is known today regarding bleeding and thrombotic disorders is based on the observations and scientific research on heparin and related drugs. The surgical, interventional, and medical usage of heparin has revolutionized medicine. For over one hundred years, new discoveries and innovative findings have continued to contribute to the expansion of our knowledge. The collection of manuscripts in this special issue of Molecules is a testimony of the continual progress in heparin sciences.

While heparin represents an old drug, the development of newer anticoagulants is primarily based on the knowledge of the mechanisms of the anticoagulant effects of heparin and related drugs. The newly developed non-vitamin K oral anticoagulants, including the anti-Xa and anti-IIa agents, along with the other mono-specific coagulation factor inhibitors, have been conceived based on the understanding of heparins’s interactions with endogenous inhibitors in plasma. These single targeting agents, which include rivaroxaban, apixaban, edoxaban, betrixaban and dabigatran are agents with specific enzyme inhibitory profiles and do not exhibit the polypharmacologic profile of heparin. The multiple pharmacologic actions of heparin have been addressed for some years, and will continue to emerge as our knowledge of vascular pathology advances.

Over the last 25 years, several major developments have occurred that have revolutionized the scientific approaches and the clinical use of heparin and related drugs. The role of some of the contributors to this volume is demonstrated by the comprehensive manuscripts written by these leading scientists.

The introduction of fractionated heparins and the subsequent development of low molecular weight heparins (LMWHs) in the 1980s has added a new dimension to the management of surgical and medical thrombosis. Eventually, with knowledge of the composition and structure of LMWHs, synthetic heparins such as the pentasaccharide were developed by French investigators. Currently, biotechnology-based methods are being employed to design and develop heparin-related drugs. The identification of the antithrombin region and subsequent synthesis of heparin-related oligosaccharides such as the pentasaccharide laid the foundation for current research programs on the development of synthetic heparin mimetics and glycomimetics with broad clinical applications.

The step-wise clinical development of heparin and related drugs from their initial indication for surgical anticoagulation onwards to medical usage and prophylactic use for post-surgical thrombosis management played a key role in the expansion of the clinical indications of heparins and LMWHs. As pleiotropic agents, heparins target multiple sites, and accordingly are relevant in the management of diverse thrombotic and vascular disorders. Additional indications are established in the areas of cardiovascular disease, cancer, autoimmune diseases, neurodegenerative diseases and sepsis-associated coagulopathy. Much of the pharmacology of heparin remains to be explored.

Heparin is the only parenteral anticoagulant that has a clinically available antagonist, protamine sulfate. Progress in identifying additional heparin neutralizing agents has been met with limited success. Approaches such as heparin digesting enzymes including heparinase, recombinant platelet factor 4, polybrene, synthetic orgamomimetics and chromatographic methods have not provided clinically favorable results. Thus, protamine sulfate is the only antagonist that is clinically used for heparin and low molecular weight heparin neutralization.

The global contaminant crisis in 2008 was a wake-up call to all of the scientists, clinicians and regulatory bodies who use and study heparin. It prompted the establishment of defined guidelines and quality assurance procedures to confirm the structural integrity and activity of these biological compounds. With the introduction of methods such as nuclear magnetic resonance (NMR) and mass spectrometry (MS), the pioneering work of Italian scientists, in particular the Ronzoni Institute (Milan, Italy), led to the establishment of analytical methods to investigate the structure and corresponding functional relevance of the components of heparins. Such methods have since been applied to the quality assurance of heparins and the detection of impurities and contaminants. Through this effort, the heparins available globally have improved purity and are safer to use. Application of chemometric techniques and holistic approaches promises to make these analytical methods even more powerful. Several of the investigators who advanced and validated these methods have contributed to this special issue.

Heparin has provided a challenging opportunity to both clinicians and scientists for innovative research that has steadily advanced the therapeutic value of this poly-therapeutic agent. Scientists from various disciplines have initially focused on improving the isolation, purification and characterization of heparin and related glycosaminoglycans. The discovery of heparin also laid the foundation for the recognition of glycosaminoglycans as being of a broad group of biologically active polymers with functional and structural heterogeneity. This dedicated issue of Molecules is a testimony to these advances.

The contributions compiled in this special issue of Molecules describe a diverse array of basic science and clinical developments. Heparins have facilitated global scientific and clinical collaborations, which are also evident in this book. Through the efforts of the scientific group at the Ronzoni Institute, under the leadership of Professor Casu, the structural and functional relationships of heparins is being established. Many of the manuscripts in this issue represent collaborative and network-based scientific work.

Advances in technology and innovations in molecular and cellular biology coupled with integrated approaches have greatly amplified research in heparin related disciplines. Our understanding of the structure and biology of heparin is far from complete, and will continue to benefit from recent advances in structural analysis, where NMR methods have played a pivotal role. One of the major areas of ongoing research relates to the correlation of structure and function, which was perceived by Professor Casu and his group to be a crucial element in the understanding of heparins.

There have been concerns regarding the supply of unfractionated heparin, which is the only anticoagulant for surgical and interventional usage. The global porcine mucosal heparin supply is primarily controlled by Chinese manufacturers. Because of the biological nature of heparin, the US Congress has expressed concern about the potential shortage of this anticoagulant and its impact on healthcare https://energycommerce.house.gov/wp-content/uploads/2018/02/20180202FDA.pdf. The need to find alternate sources for heparin has been emphasized, and the option of reintroducing bovine heparin has been underscored. Bovine heparin had been used clinically in the initial stages of the development of this drug; however, because of the concerns over BSE and potential side effects, it was withdrawn from European and US markets. However, in light of the improved manufacturing processes and South American origin of the bovine tissue, these concerns may no longer relevant. Thus, the call for bovine heparin introduction is timely and will provide a reasonable alternate for porcine heparin. Additionally through further improvement of manufacturing, the potency of bovine heparin can be enhanced to equal the porcine potency and thus can be regarded as a biosimilar product. It should be underscored that despite minor structural differences bovine heparin is clinically comparable to porcine heparin.

Sheep provide another viable source of this anticoagulant. The sheep heparin is very similar to porcine heparin and can be manufactured to exhibit a comparable biochemical and pharmacological profile. Sheep heparins have also been used in the past for clinical usage. Currently sheep heparin is being developed for clinical purposes. Pre-clinical studies have shown that sheep heparin and low molecular weight heparins are comparable to their porcine counter-parts. Thus the development of sheep heparin is also timely and will be complimentary to the development of bovine heparin.

Some of the lead initiatives in heparin research are listed below, and will potentially be the topics of future manuscripts in Molecules:

1. Improved manufacturing and quality programs for heparins and related drugs. With the advancement in purification methods and improvements in quality assurance, the heparins available today are much purer and can be defined in terms of chemical nature and biologic activities.
2. Diversification of the sources for the manufacturing of heparin. Although the currently available heparin is primarily of porcine origin, additional sources including mamallian tissues and marine sources are also being used to obtain heparin and LMWHs with comparable biologic properties to the porcine material.
3. Re-introduction of bovine heparin for clinical use is now being considered by various regulatory agencies. A dedicated monograph to detail the specifications for bovine heparin has been introduced by Brazilian regulatory agencies. The USP and other agencies are also working on specific monographs dedicated to bovine heparin.
4. Molecular and structural analyses of heparin using advanced analytical approaches have provided additional tools to understand the structural characteristics of this complex polysaccharide. Biophysical methods have been used to characterize the solution structure and molecular interaction of heparin related drugs.
5. Therapeutic profiling of non-anticoagulant heparins has been a focus of intense pre-clinical and clinical research. Novel applications of heparin and non-anticoagulant heparins include neuromodulation, immunomodulation, and cytoprotection, as well as the treatment of inflammation and cancer.
6. Biosynthetic and recombinant heparins have been developed. Utilizing hybrid chemo-enzymatic methods, heparin analogues have been synthesized by various groups. The recombinant approaches have been of limited value, but may provide enzymes and other resources to develop bioheparins.
7. Enriched heparins with higher potency have recently been developed utilizingimproved purification and site-specific sulfation approaches.
8. Development of scientific guidelines to facilitate regulatory compliance and the introduction of improved and safer products. These guidelines have primarily been developed by clinicians and scientists with working experience with heparin. Product specifications, potency designation, biosimilarity and standardization are some of the considerations that will contribute to the development of safer therapeutic products.

The contributors to this special issue of Molecules represent some of the pioneers and leaders in their area of expertise. It is hoped that this compilation will also provide an integrated forum to initiate networking and international collaboration. This collection of articles in the form of a special issue was developed under the auspices of Ronzoni Institute and Loyola University Chicago. Both of these institutions are committed to increasing the awareness of heparin and related anticoagulants for the management of thrombosis and related disorders.

